# Discoidin Domain-Containing Receptor 2 Is Present in Human Atherosclerotic Plaques and Involved in the Expression and Activity of MMP-2

**DOI:** 10.1155/2021/1010496

**Published:** 2021-12-16

**Authors:** Qi Yu, Ruihan Liu, Ying Chen, Ahmed Bilal Waqar, Fuqiang Liu, Juan Yang, Ting Lian, Guangwei Zhang, Hua Guan, Yuanyuan Cui, Cangbao Xu

**Affiliations:** ^1^Shaanxi Key Laboratory of Ischemic Cardiovascular Diseases & Shaanxi Key Laboratory of Brain Disorders, Institute of Basic and Translational Medicine, Xi'an Medical University, Xi'an 710021, China; ^2^Department of Histology and Embryology, Xi'an Medical University, Xi'an 710021, China; ^3^Department of Pathology, Zhengzhou Central Hospital, Zhengzhou 450007, China; ^4^School of Computer Science and Technology, Xi'an University of Posts and Telecommunications, Xi'an 710121, China; ^5^Faculty of Allied and Health Sciences, Imperial College of Business Studies, Lahore, Pakistan; ^6^Cardiovascular Department, Shaanxi Provincial People's Hospital, Xi'an 710010, China

## Abstract

Discoidin domain-containing receptor 2 (DDR2) has been suggested to be involved in atherosclerotic progression, but its pathological role remains unknown. Using immunochemical staining, we located and compared the expression of DDR2 in the atherosclerotic plaques of humans and various animal models. Then, siRNA was applied to knock down the expression of DDR2 in vascular smooth muscle cells (VSMCs), and the migration, proliferation, and collagen *Ι*-induced expression of matrix metalloproteinases (MMPs) were evaluated. We found that an abundance of DDR2 was present in the atherosclerotic plaques of humans and various animal models and was distributed around fatty and necrotic cores. After incubation of oxidized low-density lipoprotein (ox-LDL), DDR2 was upregulated in VSMCs in response to such a proatherosclerotic condition. Next, we found that decreased DDR2 expression in VSMCs inhibited the migration, proliferation, and collagen *Ι*-induced expression of matrix metalloproteinases (MMPs). Moreover, we found that DDR2 is strongly associated with the protein expression and activity of MMP-2, suggesting that DDR2 might play a role in the etiology of unstable plaques. Considering that DDR2 is present in the atherosclerotic plaques of humans and is associated with collagen *Ι*-induced secretion of MMP-2, the clinical role of DDR2 in cardiovascular disease should be elucidated in further experiments.

## 1. Introduction

Cardiovascular disease (CVD) is a major cause of death in the world. Atherosclerosis has been known as the common pathological basis for CVD [1]. Atherosclerotic plaque rupture primarily causes clinical events such as myocardial infarction, stroke, and thrombogenesis. Regarding previous studies, researchers suggest that the balance of macrophages and vascular smooth muscle cells (VSMCs) in the atherosclerotic plaque is a crucial factor for plaque stability. Briefly, as a result of macrophage proliferation in plaques, these inflammatory cells release many matrix metalloproteinases (MMPs) to effectively degrade extracellular matrix (ECM), such as collagens and elastin, resulting in plaque destabilization or rupture; conversely, VSMCs synthesize ECM to keep the plaque stable [2]. Despite the fact that VSMCs contribute to plaque stabilization, these cells may also promote plaque rupture by secreting MMPs during the phenotypic transition of VSMCs from the contractile to the synthetic state [3]. However, it is unknown what causes VSMCs to induce production of MMPs instead of synthesizing ECM. Notably, the major component of ECM is collagens, which account for approximately 60% of the total protein in atherosclerotic plaques [4]. These collagens not only constitute atherosclerotic plaques but also affect cell proliferation, migration, and adhesion via collagen receptors [4]. Among these collagen receptors, Discoidin domain-containing receptor 2 (DDR2) is regarded as a subgroup of tyrosine-kinase receptors, which is activated by natural ligands including collagen of types I, II, III, and X and is responsible for the communication and link between collagens and the cells [5]. DDR2 is present in various tissues, including vascular tissue and is implicated in the regulation of cell metabolism, differentiation, and growth. Interestingly, activation of DDR2 can induce production of MMPs, and therefore, DDR2 is recognized as playing a very important role in fibrosis and cancer [6]. Considering that DDR2 is found in VSMCs, an interesting question arises as to whether DDR2 also affects plaque stability via mediating MMP expression in VSMCs [7, 8]. Consequently, we designed this study to elucidate the pathological role of DDR2 in atherosclerosis.

## 2. Materials and Methods

### 2.1. Atherosclerotic Specimens and Histological Staining

Japanese white rabbits were treated by a cholesterol-rich diet containing 0.3% cholesterol and 3% corn oil for 6 or 16 weeks to induce atherosclerosis (*n* = 3, respectively), and then, rabbits were euthanized by using pentobarbital sodium at each time point. The aortic arch of each rabbit was cut into 10 cross sections (4 *μ*m) [9]. Male ApoE^–/–^ mice were euthanized by cervical dislocation. Segments of heart tissue crossing the ascending aorta and aortic sinus from male ApoE^–/–^ mice (*n* = 4) were embedded within OCT, and serial sections (8 *μ*m thick) were made as previously described [10]. Hematoxylin and eosin (H&E), oil red O, and Masson's trichrome stains were performed according to the protocols as previously described [11, 12]. Moreover, sections were performed with immunohistochemical staining against DDR2 in mouse (1 : 200; Abcam, Cambridge, UK; CST, Beverly, MA, USA) human and rabbit (1 : 200; Santa Cruz Biotechnology, Inc, Dallas, TX, USA), RAM11 of macrophages (1 : 200; Dako, CA, USA), and *α*-actin of SMC (1 : 200; Thermo Fisher Scientific, CA, USA) as previously described [11].

Human carotid plaques were collected from patients who received endarterectomy at Zhengzhou Central Hospital. Informed consents were obtained from all patients enrolled in the study, and all experiments were implemented in accordance with the guidelines and regulations set by the Ethics Committee of Xi'an Medical University (Permit No. XYJZS-201609027-1). Japanese white rabbits, SD rats, and ApoE^–/–^ mice were purchased from the laboratory animal center at Xi'an Jiaotong University (Xi'an, China). All animal experiments were performed in the animal facility of Institute of Basic and Translational Medicine at Xi'an Medical University. The animal experiments were strictly following the guidelines of animal experiment in Xi'an Medical University, which was adapted from the Guide for the Care and Use of Laboratory Animals (NIH; Bethesda, MD, USA; NIH Publication No. 85-23, revised 2011). The Laboratory Animal Administration Committee of Xi'an Medical University approved all animal experiments (Institutional Animal Care and Use Committee; Permit No. XYJZS-201608012-2).

### 2.2. VSMC Culture

VSMCs were obtained from the aortas of male SD rats (200-300 g) as previously described [13]. Cells were used in the experiments from passages 3 to 6. Before the initiation of each experiment, an additional incubation of serum-free DMEM for 24 h renders cells to be quiescent. Then, cells were exposed to oxidized low-density lipoprotein (ox-LDL) (0, 25, 50, and 100 mg/L; Yiyuan Biotechnologies, Guangzhou, China) for 24 h to simulate the proatherosclerotic condition. To activate DDR2, cells were incubated with collagen *Ι* (Sigma-Aldrich) for 48 h. To study the inhibition of signaling pathways, cells were treated with inhibitors for 30 min. SP600125 (20 *μ*mol/L; Calbiochem) is an inhibitor for JNK (c-Jun N-terminal kinase), and SB203580 (10 *μ*mol/L; Calbiochem) is an inhibitor for p38 MAPK (mitogen-activated protein kinase), and PD98059 (20 *μ*mol/L; Calbiochem) is an inhibitor for MEK (MAPK/ERK (extracellular-signal-regulated kinase) kinase). The doses of the inhibitors were referenced by the previous studies [14, 15]. Then, cells were exposed to ox-LDL (100 mg/L).

### 2.3. siRNA Interference

Small interfering RNA (siRNA) was used to knock down DDR2 expression in VSMCs as previously described [16]. Referencing a previous study, siRNA sequences were synthesized by a commercial company (sense strand, 5′- GAUGAUAGCAACACUCGGAUU-3′; antisense strand, 5′-UCCGAGUGUUGCUAUCAUCUU-3′; RiboBio, Guangzhou, China) [17]. siN05815122147 (RiboBio, Guangzhou, China) was used as a universal negative control. Transfection was performed with X-tremeGENE siRNA Transfection Reagent (Roche) and accorded to the manufacturer's instructions. Briefly, the cells were rinsed twice with phosphate-buffered saline (PBS) to reduce background interference. DDR2 siRNA with various doses (400 and 800 ng; approximately 40 and 80 pmol) were transfected into VSMCs for 6 h, and were then treated with ox-LDL (100 mg/L). The cells were collected to examine the DDR2 expression or to be performed by migration and proliferation assay.

### 2.4. Migration and Proliferation Assay

The migration of VSMCs was assessed by using the transwell permeable support insert (Corning, Lowell, MA, USA) as previously described [18]. After incubation of siRNA for 24 h without FBS, VSMCs were seeded on Matrigel (5 mg/mL; BD Biosciences, San Diego, CA, USA) of the upper compartment, and DMEM supplemented with 10% FBS was added into the lower compartment. Cells were cultured for another 24 h and then detected by crystal violet staining. Five different high-power fields per well were photographed. The positively stained VSMCs were counted by an observer blinded to the treatment protocol.

The proliferation of VSMCs was assessed by the wound-healing assay as previously described [19]. Briefly, after 24 h of siRNA treatment, a 10-l pipette tip was used to scrap an artificial wound in the monolayer across the bottom of the dish. After extensive washing, medium containing 10% FBS was removed, and cells started to migrate for the appropriate time in a 37°C incubation chamber with 5% CO_2_. At various time points, images were obtained with a Nikon TE2000 Inverted Microscope. Meanwhile, some representative dishes were performed by immunofluorescence against *α*-actin of SMC (1 : 200; Thermo Fisher Scientific, CA, USA) with Alexa Fluor 488 (1 : 200; Thermo Fisher Scientific, CA, USA). The remaining open area of the wound was quantified by using ImageJ as previously described, with some modifications [20].

### 2.5. RNA Extraction and Real-Time PCR

Total RNA was extracted from the aorta and VSMCs. Real-time PCR was performed as previously described [21, 22]. The sequences of the primers are listed in Table 1.

### 2.6. Protein Extraction and Western Blotting Analysis

Total protein was extracted from the aorta of rabbits and VSMCs as previously described [22]. The primary antibodies were against rabbit's DDR2 (1 : 500; Santa Cruz Biotechnology, Santa Cruz, CA), rat's DDR2 (1 : 500; Santa Cruz Biotechnology, Santa Cruz, CA; CST, Beverly, MA, USA), MMP-2 (1 : 500; Abcam, Cambridge, MA), TIMP-1 (1 : 500; Abcam, Cambridge, MA), TIMP-2 (1 : 500; Abcam, Cambridge, MA), p-ERK1/2 (1 : 1000; CST, Beverly, MA, USA), and GAPDH (1 : 1000; Santa Cruz Biotechnology, Santa Cruz, CA). Western blotting analysis was applied as previously described, and relative protein expression was measured by ImageJ with gel analysis [22].

### 2.7. Zymography

The supernatants along with the total protein were extracted from cultured VSMCs. Under nonreducing conditions, the equal amounts of sample protein were analyzed by SDS-PAGE in gelatin-containing acrylamide gels (2 mg/mL gelatin and 7.5% polyacrylamide) as previously described [23].

### 2.8. Statistical Analysis

All data are expressed as the mean ± SE. Two groups of comparisons were used by Student's *t*-test. Multiple groups of comparisons were performed by using one-way ANOVA with the Bonferroni test. *P* < 0.05 was considered statistically significant. The statistical calculations were performed by using SPSS 19.0 software (IBM Corp., Armonk, NY, USA).

## 3. Results

### 3.1. DDRs Are Present in Atherosclerotic Lesions of Human Being

**Figure 1:**
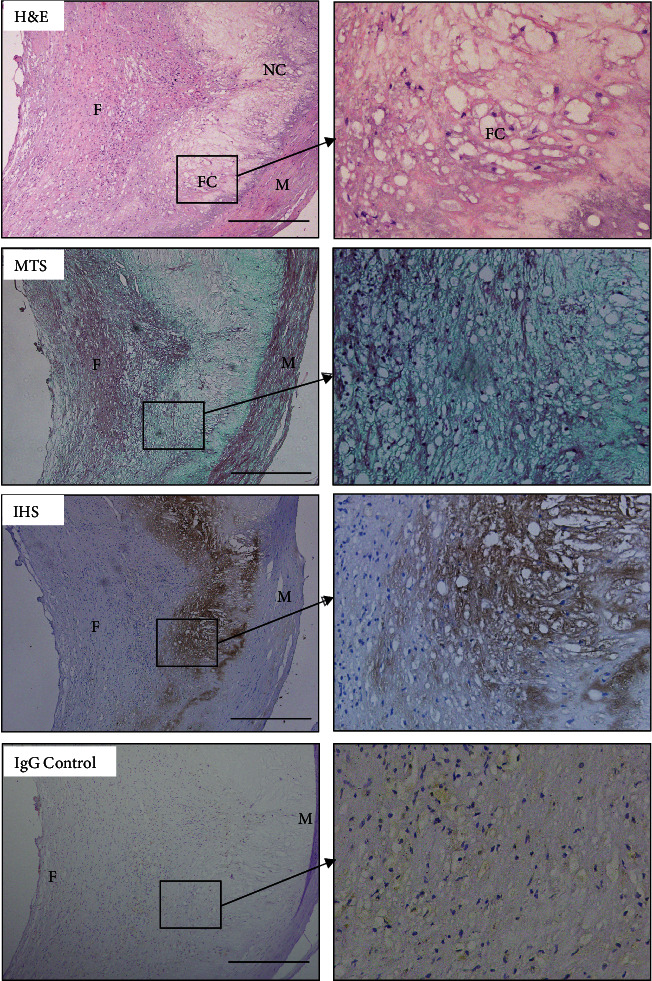
DDR2 in human carotid atherosclerotic plaque. Hematoxylin and eosin (H&E; 10x); Masson's trichrome stain (MTS; 10x); immunohistochemical staining against DDR2 (10x); rabbit IgG isotype control (10x). The area in the box is displayed as a high-power field in (b) (40x). NC: necrotic core; FC: fatty core; F: fibrous cap; M: middle membrane.

To examine whether DDR2 was present in atherosclerotic lesions, we used immunohistochemistry staining to identify DDR2 from human tissue to various animal species. Interestingly, we found expression of DDR2 in human carotid atherosclerotic plaques (Figure 1). Combined with the section with MST, we found that DDR2 distributes densely around the fatty cores of human carotid atherosclerotic plaques, and these positive stains were also adjacent to the fibrous cap and the middle membrane (Figure 1).

### 3.2. DDRs Are Present in Atherosclerotic Lesions of Animal Models

**Figure 2:**
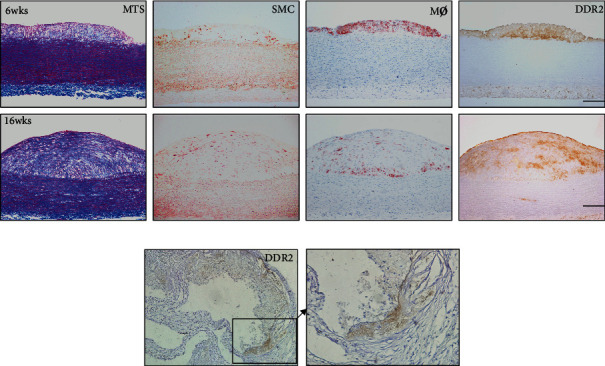
DDR2 in atherosclerotic plaques of animal models. Comparison of DDR2 expression and collagen distribution in atherosclerotic plaques of the HCD-induced rabbit model (a). Serial paraffin sections of aortic lesions were stained with Masson's trichrome stain (MTS) and immunohistochemical staining against *α*-smooth muscle actin (SMC), macrophages (M*ϕ*), and DDR2 (a) (bar = 200 *μ*m). Atherosclerotic plaques of the apoE knockout mouse model and immunohistochemical staining against DDR2 (b) (bar = 100 *μ*m). The area in the box is displayed as a high-power field on the right side (bar = 50 *μ*m).

By using a rabbit model, we attempted to investigate the relationship between DDR2 expression and atherosclerotic progression. We found that DDR2 presented in the early and middle atherosclerotic lesions of rabbits, and this receptor was located in a similar position (Figure 2(a)). In the early-stage lesion, the majority of DDR2 was deposited along the lower edge of the lesion (Figure 2(a)). In the middle-stage lesion, DDR2 was diffusely distributed in the lesion and was deposited on the surface of the lesion (Figure 2(a)). DDR2 neither apparently overlapped with the cytoplasm of macrophages nor fully overlapped with VSMCs (Figure 2(a)). To compare MTS with DDR2 staining, indicating DDR2 expression and distribution might tend to localize around collagen fibers (Figure 2(a)). To further confirm the above results, we also used immunohistochemistry staining to identify DDR2 in atherosclerotic lesions of ApoE^−/−^mice (Figure 2(b)). As Figure 2(b) shows, DDR2 was also found in atherosclerotic lesions of mice.

### 3.3. ox-LDL Upregulates DDR2 in VSMCs via the MAPK Pathway

**Figure 3:**
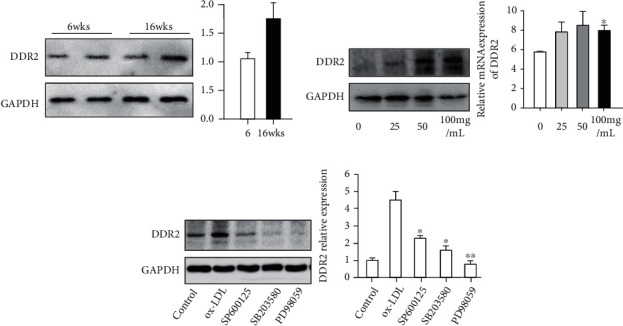
Protein expression of DDR2 in the aorta and VSMCs. Immunoblot analysis and quantification of DDR2 in aortas of HCD-induced atherosclerotic rabbits ((a); *n* = 4) and VSMCs that were incubated with various concentrations of 0, 25, 50, and 100 mg/L ox-LDL (b). Data are expressed as the mean ± standard error. ^∗^*P* < 0.05 and ^∗∗^*P* < 0.01 vs. the 0 mg group; ^##^*P* < 0.01 100 mg vs. the 25 mg group. Effect of MAPK inhibitors on ox-LDL-induced DDR2 expression (c). VSMCs were preincubated with inhibitors for 30 min and then treated with 100 ng/mL ox-LDL for 24 h in the presence of inhibitors (SP600125, SB203580, and PD98059 against JNK, MEK, and p38 MAPK, respectively). Data are expressed as the mean ± standard error. ^∗^*P* < 0.05 and ^∗∗^*P* < 0.01 vs. the ox-LDL group.

Considering that DDR2 was abundant in atherosclerotic lesions, we questioned whether DDR2 expression was associated with atherosclerotic development. To compare the protein expression of DDR2, we found that there was no significant difference between the early and middle lesions (Figure 3(a)). Next, we also questioned what caused the upregulation of DDR2 in atherosclerotic lesions. Considering ox-LDL as a key atherogenic factor, we incubated the various concentrations of ox-LDL with VSMCs for 48 h. We found that 100 mg/mL of ox-LDL significantly increased DDR2 expression in VSMCs (Figure 3(b)).

Next, we also investigated which pathway may be involved in ox-LDL-induced DDR2 expression. By using inhibitors against the MAPK pathway, we found that blocking JNK, p48, and ERK1/2 in VSMCs could neutralize ox-LDL-induced DDR2 expression, suggesting that the MAPK pathway might be responsible for the ox-LDL-induced upregulation of DDR2 (Figure 3(c)).

### 3.4. DDR2 Affects the Migration of VSMCs

**Figure 4:**
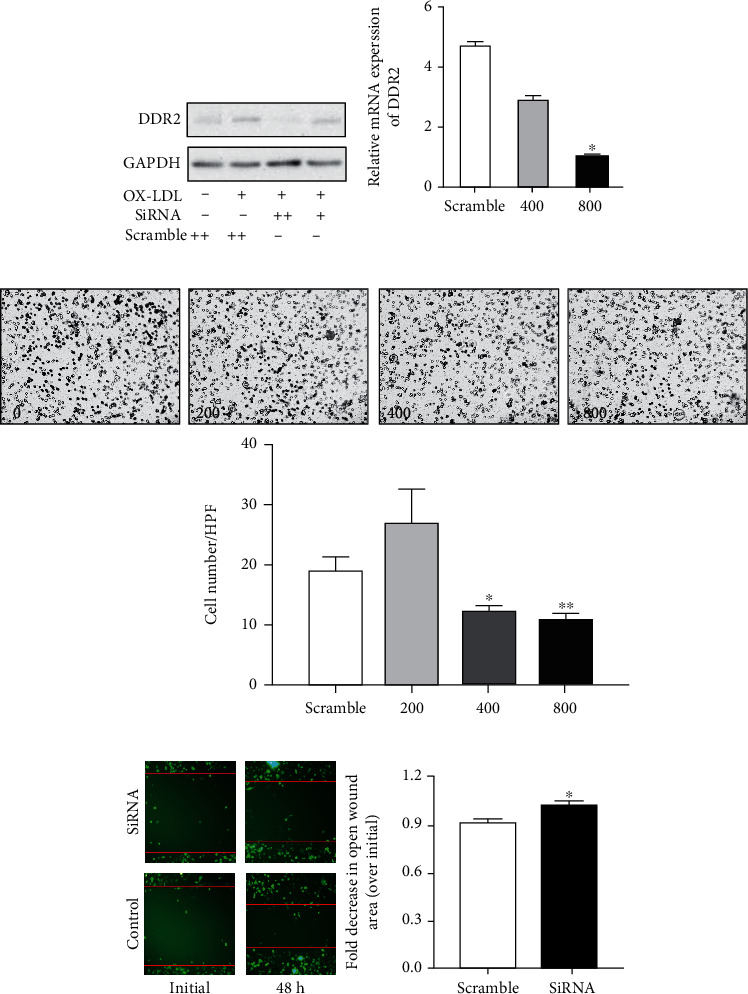
Effect of knockdown of DDR2 on the migration and proliferation of VSMCs. After 400 and 800 ng of siRNA treatments, DDR2 expression in VSMCs was analyzed and quantified by western blotting and real-time PCR (a). Migration and proliferation were assessed by transwell analysis (b) and wound healing assay (c). Data are expressed as the mean ± standard error. ^∗^*P* < 0.05 and ^∗∗^*P* < 0.01 vs. the scramble RNA-treated group.

As reported in a previous study, a specific siRNA against DDR2 was applied to suppress DDR2 protein expression. First, we used various doses of siRNA to knock down DDR2 expression in VSMCs. We found that 800 ng of siRNA could efficiently inhibit ox-LDL-induced DDR2 expression, which was quantified and confirmed with real-time PCR (Figure 4(a)). Next, to test whether inhibition of DDR2 in SMCs affected its migration, the transwell assay was performed after incubation of siRNA with VSMCs for 48 h (0, 200, 400, and 800 ng of siRNA, respectively). We found that 400 and 800 ng of siRNA reduced cell migration, indicating that the migration activity of VSMCs was inhibited by DDR2 reduction (Figure 4(b)). To confirm this result, we also used a wound healing assay to check the migration of VSMCs. As Figure 4(c) shows, we found that a decrease in DDR2 expression indeed inhibited the migration of VSMCs.

### 3.5. DDR2 Affects the mRNA Expression of MMPs in VSMCs

**Figure 5:**
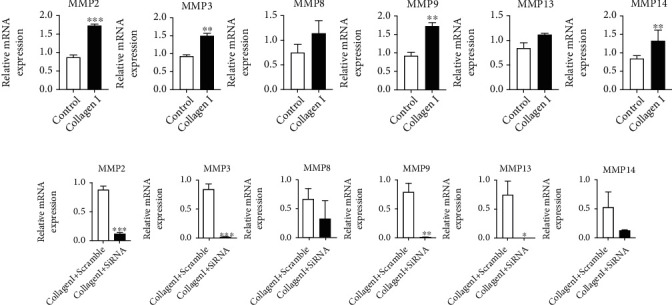
Effect of knockdown of DDR2 on mRNA expression of MMPs in VSMCs. Collagen I-induced MMP expression was assessed by real-time PCR (a). After siRNA treatment, collagen I-induced MMP expression was assessed by real-time PCR (b). Data are expressed as the mean ± standard error. ^∗^*P* < 0.05, ^∗∗^*P* < 0.01, and ^∗∗∗^*P* < 0.001 vs. the collagen I-treated group or collagen I combined with siRNA-treated group.

The invasion of VSMCs is determined by its collagenase secretion; thus, we examined whether MMP expression was reduced by DDR2 deficiency. First, we incubated collagen I with VSMCs for 48 h, and MMP (MMP-2, MMP-3, MMP-8, MMP-9, MMP-12, MMP-13, and MMP-14) expression was quantified by real-time PCR. Interestingly, we found that collagen I could upregulate MMP-2, MMP-3, MMP-9, and MMP-14 (Figure 5(a)). Next, we used 800 ng of siRNA to knock down DDR2 expression before collagen I incubation. As a result of mRNA quantification by real-time PCR, inhibition of DDR2 reversed the effect of collagen I on expression of MMPs and suppressed the mRNA expression of MMP-2, MMP-3, MMP-9, and MMP-13 (Figure 5(b)). In addition, we failed to find the mRNA expression of MMP-12 in VSMCS.

### 3.6. DDR2 Affects the Activity and Expression of MMP-2 in VSMCs

**Figure 6:**
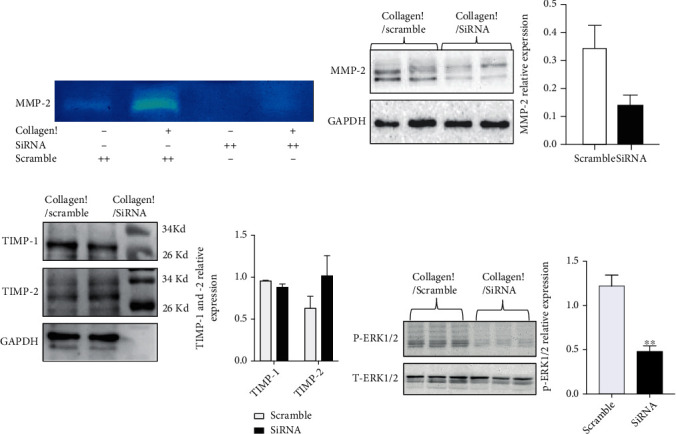
Effect of knockdown of DDR2 on the activity and expression of MMP-2 in VSMCs. The activity of MMP-2 was assessed by zymography (a). DDR2 protein expression was analyzed and quantified by western blotting (b). TIMP-1 and TIMP-2 were detected and quantified by western blotting (c). p-ERK1/2 protein expression was analyzed and quantified by western blotting (d). Data are expressed as the mean ± standard error. ^∗^*P* < 0.05 vs. the scramble RNA-treated group.

Considering that collagen I-induced mRNA expression of MMP-2 is dramatically suppressed after DDR2 deficiency, we next examined whether the inhibition of DDR2 affected the activity of MMP-2. As a result of zymography, MMP-2 activity was increased by collagen I incubation but was decreased by 800 ng of siRNA against DDR2 expression, and almost no MMP-2 activity was found without collagen I incubation before siRNA interference (Figure 6(a)). However, we did not find any bands of MMP-9 in the same zymography gel. To study whether deceased activity of MMP-2 was attributed to MMP-2 protein, we next examined MMP-2 protein expression via western blotting. Indeed, we found that MMP-2 production was decreased in the presence of siRNA against DDR2 expression (Figure 6(b)). Next, TIMP-1 and TIMP-2 were studied and quantified by western blotting, and the result showed that there was no significant difference between the two groups (Figure 6(c)). To further study the underlying mechanism, we also examined the ERK signaling according to previous report [6]. After inhibition of DDR2 by siRNA, phosphorylation of ERK1/2 was reduced (Figure 6(d)).

## 4. Discussion

Atherosclerotic plaque rupture is a serious problem for patients with cardiovascular disease, often causing unstable angina, myocardial infarction, and sudden coronary death [24]. However, how the atherosclerotic plaque becomes vulnerable remains unknown. The question remains as to why ECM tends to degrade in these unstable plaques. Notably, ECM not only constitutes atherosclerotic plaques but also activates collagen receptors to regulate the surrounding cells [25]. As a collagen receptor, DDR2 is involved with various diseases such as fibrotic diseases, arthritis, cancer, and atherosclerosis [5].

In the current study, we found that DDR2 was present in atherosclerotic plaques of various animal models, but DDR2 immunoreactivity did not totally overlap with macrophages and VSMCs. As previously described, DDR2 is highly expressed in VSMCs and also found in activated endothelial cells [26]. Based our observation, DDR2 expression is inclined to localize around collagens and fibrous caps. More importantly, for the first time, we have reported that DDR2 is highly expressed in carotid atherosclerotic plaques of human and is distributing around the fatty core of atherosclerosis and overlapping with collagen fibers. Combined to these findings, DDR2 expression in the specific regions of atherosclerotic plaque may be somehow upregulated by collagen or other proatherosclerotic factors, and the underlying mechanism needs to be addressed by the further studies. Given that ox-LDL is mainly a proatherosclerotic factor and abundant to the fat core, a question arises of whether ox-LDL can induce the expression of DDR2. Interestingly, the current research proves that ox-LDL can induce upregulation of DDR2 in a dose-dependent manner, whereas such upregulation is neutralized by the inhibition of JNK, MAPK48, and ERK, showing that the MAPK pathway may be involved with ox-LDL-induced DDR2 expression. In the vasculature, SMCs not only are responsible for the secretion of collagen fibers but also express collagen receptors to interact with ECM, regulating their own proliferation, differentiation, and migration [27]. Accordingly, we speculate that DDR2 may affect the physiological functions of VSMCs, especially when these cells are stimulated by proatherosclerotic factors. As shown in our results, VSMCs with downregulation of DDR2 display reduced differentiation and migration. Considering that VSMC infiltration in intima is a vital process of atherogenesis, DDR2 could be a mediator to regulate atherosclerotic progression [3]. Notably, given that this depends on the secretion of MMPs, another question has been raised as to whether DDR2 also regulates MMPs [28]. Indeed, MMPs can almost determine the fate of atherosclerotic plaques because MMP-mediated breakdown of ECM is a typical feature of an unstable plaque [29]. In pathological conditions, excessive ECM also stimulates VSMCs to degrade ECM as a negative feedback regulation. As a natural ligand against DDRs, collagen I can activate DDR2 in VSMCs to produce MMPs for the degradation of ECM [30]. We observed that collagen I indeed promotes VSMCs to produce various types of MMPs, especially MMP-2 and MMP-3. However, collagen I-induced expression of MMPs can be completely neutralized by knockdown of DDR2, suggesting that DDR2 plays a pivotal role in the degradation of ECM. Of note, since MMP-2 expression is dramatically affected by the knockdown of DDR2, a causal relationship between DDR2 and MMP-2 is shown in the current study. Increased MMPs do not necessarily have enzymatic activity because MMPs are not only initially synthesized as inactive state of zymogens but are also inhibited by specific endogenous inhibitors such as metalloproteinases [31]. Thus, only if DDR2 mediates the activity of MMPs can this collagen receptor be made sure to be involved with vulnerable plaques. Interestingly, decreased DDR2 indeed also inhibits the protein expression and activity of MMP-2, indicating that the effect of MMP-2 in VSMCs may proceed via collagen I-activated DDR2. With regard to the previous studies, a mechanism is suggested that collagen I causes the expression and phosphorylation of DDR2 and finally upregulates MMP-2 expression via ERK1/2 signaling [6, 32]. Accordingly, we have confirmed that phosphorylation of ERK1/2 is suppressed by inhibition of DDR2 in VSMCs, indicating that ERK1/2 may be involved with expression and activity of MMP-2. Of note, this result needs to be proven by more experiments. Moreover, we did not find pro-MMP-2, pro-MMP-9, and MMP-9 in the zymography, which did not exclude the possible regulation of activated DDR2 to other MMPs.

In conclusion, we found high expression of DDR2 in atherosclerotic plaques of human and various animal models, and DDR2 in VSMCs was involved with collagen I-induced secretion of MMP-2, suggesting that the clinical role of DDR2 in cardiovascular disease should be elucidated in further experiments. Owing to the proatherosclerotic condition, an abundance of DDR2 is present in the atherosclerotic plaques of humans and various animal models; furthermore, excessive ECM such as collagen I may also activate DDR2 to produce MMP-2 for the degradation of ECM, which is ultimately involved in the ethology of unstable atherosclerotic plaques. Once this mechanism is clarified, DDR2 may become a novel target for developing new therapeutic strategy for the control of unstable atherosclerotic plaques.

## Figures and Tables

**Table 1:** Primers were used for real-time PCR.

Gene	Forward (5′-3′)	Reverse (5′–3′)
DDR2	GATCATGTTTGAATTTGACCGA	GCACTGGGGTTCACATC
MMP-2	TTGACCAGAACACCATCG	GGTCCAGGTCAGGTGTGT
MMP-3	GCTGTGTGCTCATCCTACC	TGACAACAGGGCTACTGTC
MMP-8	AGGAATGCCACTATGATTG	CAAGAAATCACCAGAGTCG
MMP-9	ACAGCGAGACACTAAAGGC	GGCAAGTCTTCGGTGTAGC
MMP-12	GCTGGTTCGGTTGTTAGG	GTAGTTACACCCTGAGCATAC
MMP-13	ACTCAAATGGTCCCAAAC	TATCAGCAGTGCCATCAT
MMP-14	GTACCCACACACAACGCT	TTATCTGGAACACCACAGC
GAPDH	TACCCACGGCAAGTTCAACG	CACCAGCATCACCCCATTTG

## Data Availability

The data used to support the findings of this study are available from the corresponding author upon request.

## Abbreviations

DDR2: Discoidin domain-containing receptor 2
VSMCs: Smooth muscle cells
MMPs: Metalloproteinases
CVD: Cardiovascular disease
ECM: Extracellular matrix
ApoE: Apolipoprotein E
siRNA: Small interfering RNA
MAPK: Mitogen-activated protein kinase
JNK: C-Jun N-terminal kinase
MEK: Extracellular-signal-regulated kinase.
